# Efficacy and Safety of Colchicine in Prevention of Secondary Cardiovascular Outcomes Among Patients With Coronary Vessel Disease: A Meta-Analysis

**DOI:** 10.7759/cureus.26680

**Published:** 2022-07-09

**Authors:** Lubna Sattar, Rahat A Memon, Fatima Ashfaq, Syed Shah Qasim Hamdani, Rimsha Rahim Vohra, Jibran Ashraf, Baseer Khan, Noman Khurshid Ahmed, Areeba Khan

**Affiliations:** 1 Medicine, Shadan Institute of Medical Sciences, Hyderabad, IND; 2 Internal Medicine, Abington Memorial Hospital, Abington, USA; 3 Internal Medicine, Nishtar Medical University, Multan, PAK; 4 Medical College, Foundation University Medical College, Islamabad, PAK; 5 Medical College, Dow University of Health Sciences, Karachi, PAK; 6 Cardiology, National Institute of Cardiovascular Diseases, Karachi, PAK; 7 Pathology, United Medical and Dental College, Karachi, PAK; 8 Critical Care Medicine, United Medical and Dental College, Karachi, PAK

**Keywords:** cardiovascular disease, cardiovascular outcomes, meta-analysis, secondary prevention, colchicine

## Abstract

Coronary vessel disease (CVD) is a class of diseases that impacts the blood vessels and heart and is one of the leading causes of disability and death. CVD includes cerebrovascular disease and coronary heart disease, both illnesses of the vessels transporting the oxygenated blood to the brain or heart. Colchicine is an inexpensive and old drug with strong anti-inflammatory effects. Numerous randomized control trials (RCTs) have demonstrated the effectiveness of low-dose colchicine for the prevention of severe cardiovascular events without showing any signs of serious adverse effects within the regime of treatment. In the current meta-analysis, we aim to assess the efficacy and safety of colchicine for secondary cardiovascular outcome prevention among patients with clinically proven CVD. The current meta-analysis was carried out using the preferred reporting items for systematic reviews and meta-analysis (PRISMA) guidelines. PUBMED, Cochrane, and EMBASE databases were used to search for RCTs comparing colchicine and placebos for the prevention of secondary cardiovascular outcomes. The primary efficacy endpoint was mortality due to cardiovascular disease, stroke, urgent coronary revascularization, and myocardial infarction. Secondary efficacy outcomes included death due to all-cause mortality. Seven RCTs were reviewed, with a pooled sample size of 12114, out of which 6099 were randomized to the colchicine group, and 6015 were randomized to the control group. The decrease in cardiovascular events, including myocardial infarction, stroke, urgent coronary revascularization, and cardiac-related death, was significantly lower in patients randomized to colchicine (p-value<0.05). The incidence of safety outcomes did not vary significantly different between groups (p>0.05). In patients with CVD, compared to standard medical therapy, colchicine significantly decreases the risk of cardiovascular events such as cardiovascular-related death, myocardial infarction, stroke, and urgent coronary revascularizations.

## Introduction and background

Coronary vessel disease (CVD) is one of the leading causes of disability and death [1]. CVD is a class of diseases that impacts the blood vessels and heart. It includes cerebrovascular disease and coronary heart disease, both illnesses of the vessels transporting the oxygenated blood to the brain or heart [2]. One of the vital pathophysiological mechanisms of CVD is the development of plaques, also known as atherosclerotic lesions [3]. These lesions can result in organ damage, such as heart failure, as well as chronic ischemia. But these plaques can potentially burst, leading to sudden thrombotic events like strokes or myocardial infarctions [4]. Inflammatory processes appear to have a significant part in the mechanisms that cause atherosclerotic plaques to become unstable and then rupture [2,4].

The suggested treatment for atherosclerosis and coronary artery disease (CAD) in clinical guidelines reflects the complexity of the condition [5]. The comprehensive approach to patient care combines lifestyle changes with medication interventions, such as lipid-lowering therapy, blood pressure management, glucose control, and antithrombotic therapy [4]. These treatments have been shown as effective at slowing the development of atherosclerosis or drastically lowering the frequency of cardiovascular outcomes [5]. Inflammation has become a significant therapeutic target in individuals with atherosclerosis in recent times [6]. This therapeutic impact was more noticeable in patients who successfully reduced their inflammatory indicators. As a result, cardiovascular events because of plaque destabilization and rupture were decreased [7-8].

Colchicine is an inexpensive and old drug with strong anti-inflammatory effects. It has been used for the treatment of different diseases such as gout, pericarditis, primary biliary cirrhosis, Behçet's disease, and familial Mediterranean fever [9]. Besides this, colchicine is a comparatively safe and low-cost drug that has been available for several centuries [10]. Numerous randomized control trials (RCTs) have demonstrated the effectiveness of low-dose colchicine for the prevention of severe cardiovascular events without showing any signs of serious adverse effects within the regime of treatment [11-12]. Colchicine may hinder the inflammatory mechanism causing the destabilization or development of atherosclerotic plaques. Colchicine is also associated with the reduction of high-sensitivity C-reactive protein in individuals with CAD [13].

In recent times, the role of colchicine in the prevention of secondary cardiovascular events has been the focus of extensive research. In the current meta-analysis, we aim to assess the efficacy and safety of colchicine for secondary cardiovascular outcome prevention among patients with clinically proven CVD.

## Review

Methodology

The current meta-analysis was carried out using the preferred reporting items for systematic reviews and meta-analysis (PRISMA) guidelines. PUBMED, Cochrane, and EMBASE databases were used to search for RCTs comparing colchicine and placebo for the prevention of secondary cardiovascular outcomes. Key terms used for searching for relevant articles included “colchicine” “coronary artery disease”, “cardiovascular outcomes”, “secondary prevention” and “cardiovascular disease”.

Two reviewers independently screened titles and abstracts of all the potential studies for inclusion. Any disagreement between the two reviewers was resolved through discussion or with the consensus of the third reviewer. Full-text was retrieved from all relevant articles, and two reviewers independently screened the full-texts articles to assess for inclusion criteria.

Articles that fulfilled the following criteria were included in the current meta-analysis: 1) the study compared colchicine to placebo, no colchicine or standard management for prevention of secondary cardiovascular outcomes 2) the study reported at least one cardiovascular outcome, 3) the study followed up patients to at least one month. Studies that were published in a language other than English were excluded. Besides this, studies were excluded if they compared one dose of colchicine with another dose. No restrictions were placed on the time and place of publication.

From the eligible articles, data on study characteristics such as first author, year of publication, sample size, intervention, the mean age of participants, major inclusion criteria, and median follow-up period were extracted and presented in the form of a table.

Assessment of risk of bias

Two authors assessed the risk of bias independently for each included study utilizing the criteria defined in the Cochrane Handbook for Systematic Reviews of Interventions. Any disagreement between the two reviewers was resolved through discussion or with the consensus of another review author. Six domains were assessed to evaluate the risk of bias, including random sequence generation, allocation concealment, blinding of personnel and participants, blinding of outcomes assessment, incomplete outcome data, selective outcome reporting, and other biases.

Outcome assessment

The primary efficacy endpoint was mortality due to cardiovascular disease, stroke, urgent coronary revascularization, and myocardial infarction. Secondary efficacy outcomes included death due to all-cause mortality. Safety outcomes were assessed by comparing the adverse events and serious adverse events such as gastrointestinal events, infection, cancer, pneumonia, and other adverse events between the colchicine group and control group.

Statistical analysis

Analysis was performed using Cochrane Review Manager, version 5.4 (Cochrane, London, United Kingdom). For dichotomous data, pooled risk ratios (RR) were calculated along with a 95% confidence interval (CI) using the Mantel Haenszel method. Heterogeneity analysis was performed by calculating the I2 value. If the value of I2 is less than 50, data was considered homogenous and analyzed using a fixed-effect model. Otherwise, a random effect model was chosen. Forest plots were produced to show the relative effect size of colchicine versus the control group on efficacy and safety outcomes. A p-value of 0.05 or less was considered statistically significant.

Results

**Figure 1:**
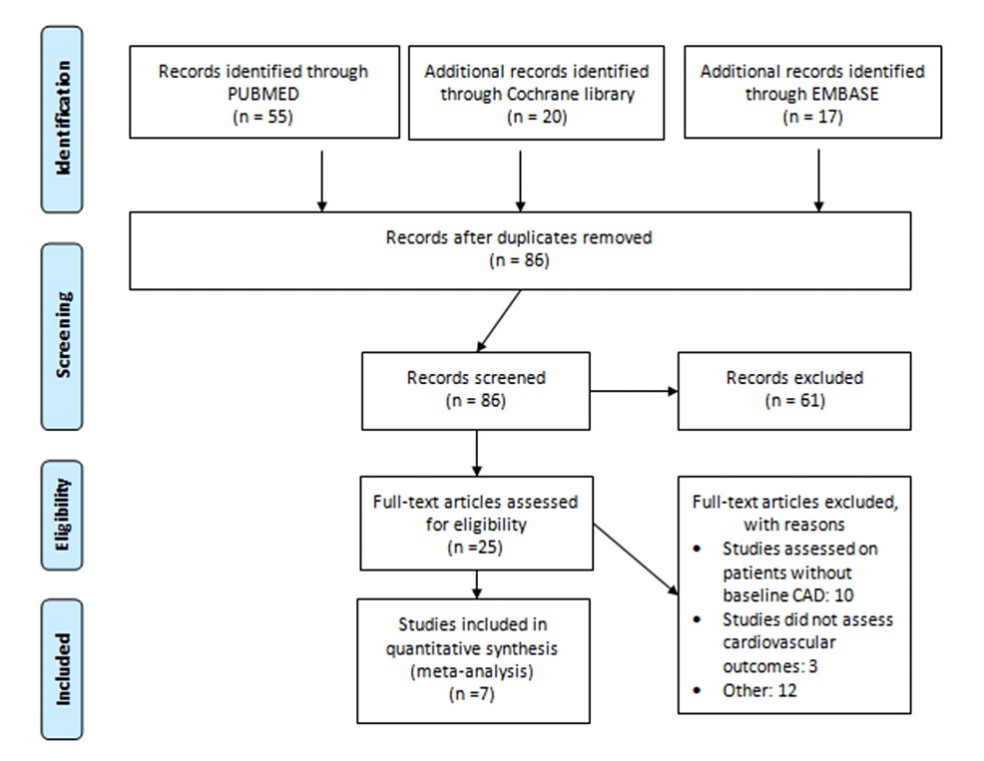
PRISMA flow diagram PRISMA: Preferred Reporting Items for Systematic Reviews and Meta-Analyses

Through systematic search, 92 studies were identified, as shown in Figure 1. After removing duplicates, the title and abstract of 86 studies were screened. Only 25 studies were eligible for full-text screening. Of these, only seven studies were used in the current meta-analysis [10-12,14-17]. Characteristics of all included studies are shown in Table 1.

The pooled sample size of enrolled patients was 12,114, out of which 6099 were randomized to the colchicine group and 6015 were randomized to the control group. The control group was either placebo or no medicine or standard care. Four studies had a follow-up period of 12 months or more [10-12,14] while three studies had a follow-up time of less than 12 months [15-17]. Among all included studies, three studies were multi-centered [10,12,14].

**Table 1:** Characteristics of included studies ACS: acute coronary syndrome; PCI: percutaneous coronary intervention; CAD: coronary artery disease

Author	Publication Year	Inclusion Criteria	Groups	Sample Size	Mean age	Multicenter (Yes/No)	Median follow-up time
Akodad et al. [17]	2017	Patients with ACS and treated with PCI	Colchicine 1 mg for one month	23	59.9 years	No	One month
			Conventional treatment	21			
Deftereos et al. [15]	2013	Patients with CVD	0.5 mg twice daily for six months	140	66.7 Years	No	Six months
			Placebo	139			
Nidorf et al. [11]	2013	Patients with angiographically proven coronary disease	colchicine 0.5 mg once daily	282	67.5 Years	No	24 months
			Placebo	250			
Nidorf et al. [12]	2020	Patients with coronary disease	colchicine 0.5 mg once daily	2762	65.8 Years	Yes	28.6 months
			Placebo	2760			
O Keefe et al [16]	1992	Patients with ACS undergoing PCI in a coronary artery	Colchicine 0.6 mg for six months	130	60.5 Years	No	Six months
			Placebo	67			
Tardif et al. [10]	2019	Patients having myocardial infarction within 30 days of enrollment	Colchicine 0.5 mg once daily	2366	60.5 Years	Yes	22.6 months
			Placebo	2379			
Tong et al. [14]	2020	Adults presented with ACS and had evidence of CAD	0.5 mg oral colchicine twice daily for the first month, followed by 0.5 mg daily for 11 months	396	59.8 years	Yes	12 months
			Placebo	399			

Risk of bias in included studies

**Figure 2:**
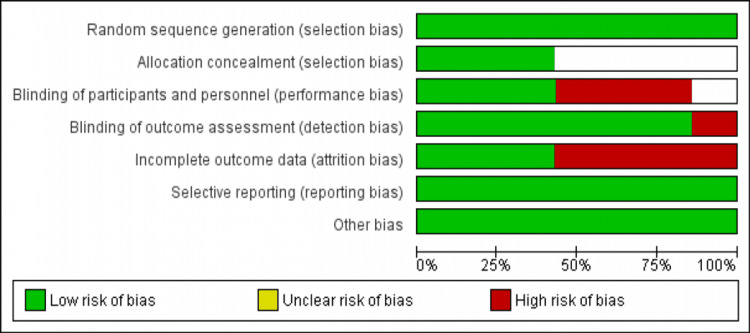
Risk of bias graph

The overall risk of bias was moderate. No indication of selection bias has been found in any of the studies, but allocation concealment was not clear in three out of seven studies. Blinded outcome assessment was frequently low, and only one study had a high blinded outcome assessment. Overall, the quality of the study was moderate. Figure 2 shows the risk of bias plot.

Effects of colchicine on secondary cardiovascular events

**Figure 3:**
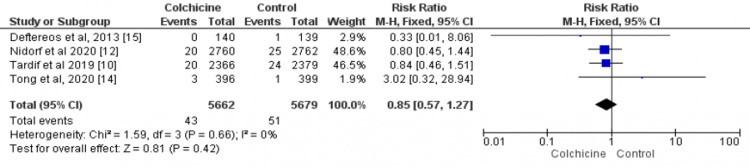
Forest plot for cardiac-related death The pooled RRs with 95%CI were calculated using fixed-effects models. Weight refers to the contribution of each study to the overall pooled estimate of treatment effect. Each square and horizontal line denotes the point estimate and 95%CI for each trial’s RR. The diamonds signify the pooled RR; the diamond’s center denotes the point estimate and the width denotes the 95%CI RR: risk ratio

Data on the incidence of cardiovascular-related deaths were available for four studies [10,12,14-15], including 11,341 patients (5662 in the colchicine group and 5679 in the control group). Overall, 43 deaths were reported in the colchicine group and 51 deaths were reported in the control group, and the difference is insignificant between the two groups (RR: 0.85; 95%CI: 0.57-1.27) as shown in Figure 3. There was insignificant heterogeneity between the results of included studies (I² = 0%).

**Figure 4:**
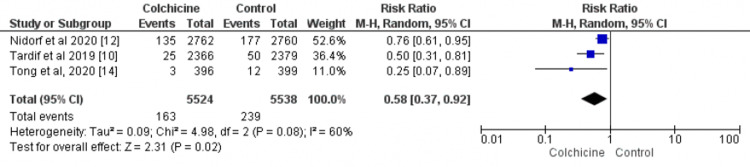
Forest plot for urgent coronary revascularization The pooled RRs with 95%CI were calculated using random-effects models. Weight refers to the contribution of each study to the overall pooled estimate of treatment effect. Each square and horizontal line denotes the point estimate and 95%CI for each trial’s RR. The diamonds signify the pooled RR; the diamond’s center denotes the point estimate, and the width denotes the 95%CI RR: risk ratio

Data on urgent coronary revascularization were available in three studies [10,12,14]. A total of 11,062 patients were enrolled in these three RCTs (5524 in the colchicine group and 5538 in the control group). The risk of urgent coronary revascularization is 42% lower in the colchicine group as compared to the control group (RR: 0.58; 95%CI: 0.37-0.92), as shown in Figure 4. There was high between‐study heterogeneity (I² = 60%).

**Figure 5:**
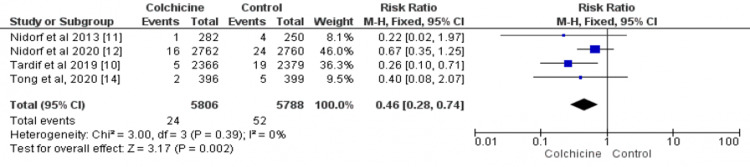
Forest plot for stroke The pooled RRs with 95%CI were calculated using fixed-effects models. Weight refers to the contribution of each study to the overall pooled estimate of treatment effect. Each square and horizontal line denotes the point estimate and 95%CI for each trial’s RR. The diamonds signify the pooled RR; the diamond’s center denotes the point estimate, and the width denotes the 95%CI RR: risk ratio

Data on stroke were available in four studies [10-13,14], including 11,594 patients (5806 in the colchicine group and 5788 in the control group). The risk of stroke was significantly lower in the colchicine group as compared to the control group (RR: 0.46; 95%CI: 0.28-0.74), as shown in Figure 5. There was insignificant heterogeneity between the results of included studies (I² = 0%).

**Figure 6:**
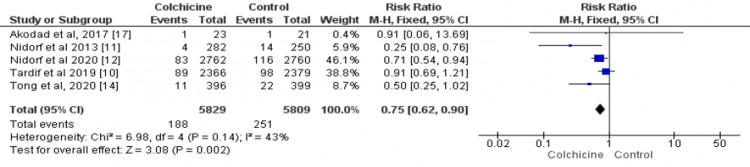
Forest plot for myocardial infarction The pooled RRs with 95%CI were calculated using fixed-effects models. Weight refers to the contribution of each study to the overall pooled estimate of treatment effect. Each square and horizontal line denotes the point estimate and 95%CI for each trial’s RR. The diamonds signify the pooled RR; the diamond’s center denotes the point estimate, and the width denotes the 95%CI RR: risk ratio

Data on the incidence of myocardial infarction were available for five studies [10-12,14,17], including 11,640 patients (5829 in the colchicine group and 5811 in the control group). The risk of myocardial infarction is 25% lower in the colchicine group as compared to the control group (RR: 0.75; 95%CI: 0.62-0.90), as shown in Figure 6. There was insignificant heterogeneity between the results of included studies (I² = 43%).

**Figure 7:**
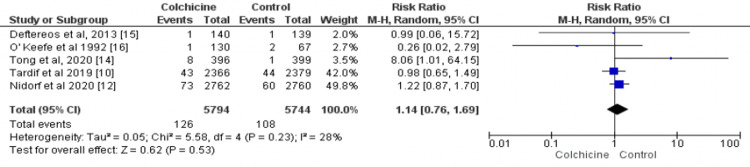
Forest plot for all-cause mortality The pooled RRs with 95%CI were calculated using fixed-effects models. Weight refers to the contribution of each study to the overall pooled estimate of treatment effect. Each square and horizontal line denotes the point estimate and 95%CI for each trial’s RR. The diamonds signify the pooled RR; the diamond’s center denotes the point estimate, and the width denotes the 95%CI RR: risk ratio

Data on all‐cause mortality were available for five studies [10,12,14-16], including 11,538 patients (5794 in the colchicine group and 5744 in the control group). The relative risk of all-cause mortality was 1.14 (95%CI: 0.76-1.69), as shown in Figure 7. Heterogeneity between studies was low (I² = 28%).

In sensitivity analysis of studies that included acute coronory syndrome (ACS) patients, the urgent coronary revascularization was significantly lower in the colchicine group as compared to the control group (RR: 0.45; 95%CI: 0.29-0.71), and heterogeneity reduced from 60% to 2%. Similarly, myocardial infarction was significantly lower in the colchicine group as compared to the control group (RR: 0.68; 95%CI: 0.53-0.88), and heterogeneity reduced from 43% to 0%. Table 2 shows the sensitivity analysis, and the results are similar to the overall analysis.

**Table 2:** Results of sensitivity analysis RR: risk ratio *Significant at p-value<0.05

Outcome	Studies	RR (95% CI)	Heterogeneity
Cardiac-related death	[10,14]	0.93 (0.53-163)	0%
Urgent coronary revascularization	[10,14]	0.45 (0.29-0.71)*	2%
Stroke	[10,14]	0.29 (0.13-0.68)*	0%
Myocardial infarction	[10,14,17]	0.68 (0.53-0.88)*	0%

Safety analysis

The adverse events related to study medication or placebo reported by individual studies are shown in Table 3. No significant differences were reported in any of the adverse events between the colchicine and control groups as the p-value is more than 0.05. In both groups, the most common adverse events were gastrointestinal, being 15.68% and 14.30% in the colchicine group and control group, respectively.

**Table 3:** Adverse events RR: risk ratio

Adverse Event	Colchicine n (%)	Control n (%)	RR (95% CI)
Gastrointestinal events	881 (15.68)	797 (14.30)	1.15 (0.94-1.41)
Cancer	163 (3.20)	168 (3.29)	0.97 (0.79-1.20)
Infection	188 (3.69)	182 (3.56)	0.77 (0.46-1.29)
Pneumonia	67 (1.31)	64 (1.25)	1.32 (0.48-6.63)
Others	64 (1.13)	143 (2.56)	0.47 (0.20-1.10)

Discussion

The current meta-analysis was conducted to determine the efficacy and safety of colchicine for secondary cardiovascular outcome prevention among patients with clinically proven CVD. The current meta-analysis included seven RCTs, including 12,114 patients, out of which 6099 were randomized to the colchicine group, and 6015 were randomized to the control group with a median follow-up up to 28.6 months. The current meta-analysis found that the incidence of major cardiovascular events such as myocardial infarction, stroke, cardiac-related death, and urgent coronary revascularization was lower in the colchicine group as compared to the control group. In addition, colchicine has a favorable safety profile. Pooling RR from RCTs showed that the risk of major cardiovascular events was attributed to a risk decrease of 58%, 54%, and 25% for urgent coronary revascularization, ischemic strokes, and myocardial infarction, respectively. Pooled results showed that no significant difference is there in the incidence of all-cause mortality with colchicine between patients who received colchicine and patients in the control group.

Colchicine appears to have positive benefits in the prevention of cardiac disease, and plausible explanations for these effects have been put up. Colchicine has a broad anti-inflammatory impact [14]. It does not only target the NLRP3 inflammasome, whose activation causes the upregulation of IL-1 and IL-6 downstream but also disrupts microtubules and has antimitotic effects [18]. As cholesterol crystals within atherosclerotic plaques are known to cause local inflammation and provoke plaque instability, it is believed that colchicine may also be beneficial in treating cardiovascular illness through the suppression of these crystals [19].

Several observational studies have been conducted on the utilization of colchicine for the prevention of cardiovascular outcomes. These studies are limited to the prevention of CAD in patients with gout, liver disease, and without CAD at baseline [20-22]. The lower risk of cardiovascular outcomes in a low-risk population further supports the role of colchicine in preventing the advancement of atherosclerosis and plaque destabilization, even though final conclusions cannot be derived from such observational research [22].

The utilization of colchicine for the prevention of primary or secondary cardiovascular outcomes will need longer terms to use. Based on experience treating familial Mediterranean fever, it appears that there are few cases of late negative effects from very long-term continuous colchicine doses of up to 2 to 3 mg per day [23-24]. Long-term researches in individuals with cardiovascular illness at baseline are important in proving the safety of long-term lower dose therapy in this population. Among all the studies used in the current meta-analysis, four studies had a follow-up period of 12 months or more, while three studies had a follow-up time of fewer than 12 months.

Colchicine is prescribed widely, and it has a known side-effect and safety profile. It is considered a low-cost drug. In a meta-analysis conducted by Verma et al., including seven RCTs involving colchicine, the relative risk of discontinuation was because side effects was 4.34 (95% CI, 1.7-11.07) as compared to the placebo [25]. In the current meta-analysis, the most common adverse effects were gastrointestinal, but no significant differences were there between the gastrointestinal events in patients who received colchicine and those who got placebos. The meta-analysis conducted by Siak et al. included nine RCTs and also reported that the most common adverse effect is gastrointestinal symptoms such as vomiting, nausea, and diarrhea in up to 20% of the patients [26].

The current meta-analysis has certain limitations. Firstly, heterogeneity was there between the study populations enrolled in each of the trials that may describe the risk differences in cardiovascular outcomes between studies. In the current analysis, we tried to minimize the heterogeneity by performing a sensitivity analysis, in which studies involving ACS patients only were included. Furthermore, there were a limited number of studies; therefore, a statistical test for homogeneity may not have been optimal.

## Conclusions

In patients with CVD, colchicine to standard medical therapy significantly decreases the risk of cardiovascular events such as myocardial infarction, stroke, and urgent coronary revascularizations. Even though the clinical benefit of colchicine in the prevention of secondary cardiovascular events has been shown in the current meta-analysis, exciting new data show its potential advantage for the prevention of secondary vascular outcomes in patients with clinically proven CVD. This advantage raises questions about the role of inflammation in both coronary disease and other inflammatory and immunomodulatory pharmacotherapies, which opens up new research directions.
